# The COVID-19 Study of Healthcare and Support Personnel (CHAMPS): Protocol for a Longitudinal Observational Study

**DOI:** 10.2196/30757

**Published:** 2021-10-07

**Authors:** Peter G Kaufmann, Donna S Havens, Janell L Mensinger, Patricia K Bradley, Heather M Brom, Linda C Copel, Alexander Costello, Christine D'Annunzio, Jennifer Dean Durning, Linda Maldonado, Ann Barrow McKenzie, Suzanne C Smeltzer, Jennifer Yost, CHAMPS Study Investigators

**Affiliations:** 1 M. Louise Fitzapatrick College of Nursing Villanova University Villanova, PA United States; 2 Department of Mathematics and Statistics Villanova University Villanova, PA United States; 3 Villanova University Villanova, PA United States

**Keywords:** COVID-19, SARS-CoV-2, stress, depression, anxiety, sleep, social support, resilience, mental health, physical health

## Abstract

**Background:**

Early in the development of the COVID-19 pandemic, it was evident that health care workers, first responders, and other essential workers would face significant stress and workplace demands related to equipment shortages and rapidly growing infections in the general population. Although the effects of other sources of stress on health have been documented, the effects of these unique conditions of the COVID-19 pandemic on the long-term health and well-being of the health care workforce are not known.

**Objective:**

The COVID-19 Study of Healthcare and Support Personnel (CHAMPS) was designed to document early and longitudinal effects of the pandemic on the mental and physical health of essential workers engaged in health care. We will investigate mediators and moderators of these effects and evaluate the influence of exposure to stress, including morbidity and mortality, over time. We will also examine the effect of protective factors and resilience on health outcomes.

**Methods:**

The study cohort is a convenience sample recruited nationally through communities, professional organizations, networks, social media, and snowball sampling. Recruitment took place for 13 months to obtain an estimated sample of 2762 adults who provided self-reported information administered on the web through structured questionnaires about their work environment, mental and physical health, and psychosocial factors. Follow-up questionnaires will be administered after 6 months and annually thereafter to ascertain changes in health, well-being, and lifestyle. Participants who consented to be recontacted form the longitudinal cohort and the CHAMPS Registry may be contacted to ascertain their interest in ancillary studies for which they may be eligible.

**Results:**

The study was approved by the Institutional Review Board and launched in May 2020, with grants from Travere Therapeutics Inc, McKesson Corporation, anonymous donors, and internal funding from the M. Louise Fitzpatrick College of Nursing at Villanova University. Recruitment ended in June 2021 after enrolling 2762 participants, 1534 of whom agreed to participate in the longitudinal study and the registry as well as to be contacted about eligibility for future studies.

**Conclusions:**

The CHAMPS Study and Registry will enable the acquisition of detailed data on the effects of extended psychosocial and workplace stress on morbidity and mortality and serve as a platform for ancillary studies related to the COVID-19 pandemic.

**Trial Registration:**

ClinicalTrials.gov NCT04370821; https://clinicaltrials.gov/ct2/show/NCT04370821

**International Registered Report Identifier (IRRID):**

DERR1-10.2196/30757

## Introduction

### Background

Globally, SARS-CoV-2 has infected more than 224 million individuals, with more than 41 million cases in the United States [1]. The first report that a new coronavirus was the cause of a spate of pneumonia cases in Wuhan, China, was issued in early January 2020 by the World Health Organization, and the first case in the United States was reported two weeks later [2]. By early spring of 2020, infections had risen dramatically in several countries, and projections suggested that the pandemic could impose severe strains on the ability of US hospitals to deliver care [3]. Experience from the severe acute respiratory syndrome (SARS) outbreak in 2003 [4,5] suggested that because of its heavy burden on the health care system, the rapidly rising number of COVID-19 cases would have a large impact on the mental and physical health of the health care workforce. Such concerns indeed began to emerge early in the spring of 2020 [6]. It also seemed reasonable to expect that laboratory workers, office personnel, first responders, and others who were engaged less directly in caring for patients with COVID-19 as compared to health care workers, would experience similar strains on their psychological well-being. First responders, including police, fire, and emergency medical services, may experience higher rates of exposure to SARS-CoV-2 than the general population [7].

Health care systems in numerous countries were overwhelmed by the COVID-19 pandemic, which has caused increased pressure on frontline health workers [8]. Hospitals in New York City**,** for example, have had to reconfigure patient-care spaces and restructure clinical teams rapidly to address the increase in the number of patients with COVID-19 [9]. The overwhelming workload, increasing numbers of suspected and confirmed COVID-19 cases, lack of evidence-based treatments, shortages of personal protective equipment (PPE), extensive media coverage, and perceptions of inadequate support may contribute to the mental burden of health care workers [10].

Concerns of health care workers that they might not only become infected with COVID-19 but also transmit it to family members and friends add to their psychological burden [9]. The combined result is that the COVID-19 pandemic can be expected to have a prolonged impact on the mental health of a broad range of workers who are essential for delivering health care [11,12].

### Rationale

In the context of the COVID-19 pandemic, we anticipated that health care workers would face anxiety and depressive symptoms due to traumatic patient-care experiences and the risk of SARS-CoV-2 infection to family, friends, and colleagues [13]. Frontline health care workers were among the most vulnerable groups at risk of mental health issues during the early phases of the COVID-19 pandemic; however, the numerous risks to the well-being of health care workers remain poorly understood [14]. High prevalence of anxiety and depressive symptoms among frontline health care workers caring for patients with COVID-19 has been reported [14], but their duration and impact on future physical health are not known. The risks to the mental well-being of health care workers are likely multifaceted, with a dearth of information regarding who might benefit from preventive interventions [15].

Even under normal circumstances, workplace stress has effects on physical health [16], and these effects increase as decision latitude and control over work decrease [17]. With rapidly increasing caseloads in health care facilities, the COVID-19 pandemic seemed ideally constituted to create a high-demand, low-decision latitude environment. Added to this was the likelihood of a high proportion of adverse outcomes and mortality of patients due to the absence of effective treatments in the first months of the pandemic. Finally, PPE shortages [9,14,18] increased the risk of infection for health care workers and those with whom they have a close relationship, including family, household members, and others, which has the potential to add to other sources of stress.

Emotional stress and stressful life events have been shown to contribute to the six leading causes of death in the United States—cancer, coronary artery disease, accidents (unintentional injuries), respiratory disorders, cirrhosis of the liver, and suicide—as well as to type 2 diabetes, sleep disorders, and other health conditions [19]. Workplace stress in particular has been associated with hypertension [20] and coronary heart disease [21]. High workplace demands and low decision latitude, combined with low rewards, are prospective risk factors for common mental disorders [22]. The effects of stressful working conditions on the short-term mental health of health care personnel during this pandemic have already begun to emerge [9,18].

The effects of stress on long-term physical and mental health can take many years to become manifest. Military combat deployment [23,24] and elevated symptoms of posttraumatic stress disorder (PTSD) in civilians have been associated with increased risk of hypertension, myocardial infarction, and stroke [25,26]. Longitudinal trends show that the likelihood of developing multiple physical symptoms over time is higher for those who were deployed in combat than those without combat experience [27].

Research on the effects of stress on the COVID-19 workforce [9,18] is underway but is in its early stages [28], with mixed results [29]. Some studies have noted the potential importance of resilience in response to stress, including in health care workers [30], but adequate supporting data on the role of resilience in response to stress are lacking [31]. The COVID-19 pandemic experience of essential workers provides an opportunity to develop new information on the influence of sustained stress on the short- and long-term physical and mental health of the workforce involved in providing health care or to support health care personnel, as well as on the potential influence of aspects of resilience and its role in moderating the effects of stress.

Based on the extensive body of literature on the psychosocial influences on health, it is likely that the COVID-19 pandemic will have deleterious effects on the health of health care workers and support staff in a broad range of professions [32]. Over time, as COVID-19 treatments and management improve and infection rates decrease through public health measures and vaccination, we anticipate that sources of stress will decrease, resulting in a gradient of influence on health among health care workers who enroll in the COVID-19 Study of Healthcare and Support Personnel **(**CHAMPS) during later phases of the pandemic. The CHAMPS Study and Registry is designed to ascertain the time course and magnitude of these changes on the long-term mental and physical health of essential workers in the health care environment.

### Objectives

The objective of the CHAMPS Study and Registry is to assess the short- and long-term physical, social, and behavioral health of personnel (“essential workers”) involved in supporting or delivering care for patients with COVID-19. Included are first responders, maintenance and support staff working in clinical settings, and health care professionals of all specialties and services. At enrollment, baseline data were obtained on the study participants’ working environment, lifestyle, and mental and physical health. Subsequent waves of data collection will obtain follow-up information after 6 months and then annually through 2024. The study was approved by the Villanova University Institutional Review Board (IRB) in May 2020.

The primary objectives of the CHAMPS study are to (1) determine the extent to which working in health care affects future physical and mental health; (2) evaluate trends over the time course of the evolving pandemic; (3) identify exposure variables that are associated with incident changes in health over time; and (4) identify factors that influence short- and long-term health. Specific hypotheses include the following:

1. Measures of mental health symptoms of anxiety, traumatic stress, depression, insomnia, disordered eating, resilience, burnout, and poor self-reported health will be more prevalent in essential workers than in the general population and greater in magnitude than reported in similar populations before the COVID-19 pandemic.
2. The severity of mental health symptoms will be associated with prevalence of SARS-CoV-2 infection rates in the geographic region in which the study participants work.
3. Mental health symptoms will persist longer among study participants with more severe symptoms.
4. Essential workers who report elevated symptoms of anxiety, traumatic stress, or depression will experience exacerbation of existing physical health conditions over time and higher incidence of new physical health conditions.
5. Severity and progression of mental and physical health conditions will be exacerbated by the degree of perceived exposure to adverse working environments, such as availability of personal protective equipment, extended working hours, and adequacy of staffing levels.

## Methods

### Study Components

CHAMPS consists of three components: (1) a cross-sectional study of respondents who consented to participate in a single assessment of health; (2) a longitudinal study of participants who consented to repeated waves of data collection; and (3) a registry comprised of participants who enrolled in the longitudinal study. The registry serves as a source of study participants who may be eligible for future ancillary observational studies as well as randomized clinical trials.

### Questionnaires

To address the specific hypotheses, we selected validated instruments available for mental and physical health variables of interest. They are described in detail in the *Measures* section. Demographic, occupation, work environment, and geographic questions were written by professionals who work in health care.

### Participants and Recruitment

Eligible participants were adult essential workers involved in health care. For the purposes of the CHAMPS study, essential workers were defined as adult (aged 18 years or older) health care personnel, support personnel, and first responders working in any health care facility or in the community. Included were those working in facilities that are involved in patient care, such as hospitals, clinics, private practices, or screening facilities; those directly involved in patient care (eg, physicians, nurses, phlebotomists, respiratory therapists, pharmacists); laboratory staff; service employees (office staff, maintenance, housekeeping, food service); individuals working in long-term care facilities; and first responders (eg, police officers, firefighters, emergency medical technicians, paramedics). Collectively, we refer to these as “essential workers.” Enrollment took place over 13 months, from May 2020 through June 2021, throughout the United States.

Recruitment included advertising through local and national professional and trade organizations, news, social and professional media, alumni organizations, and word of mouth to describe the study and disseminate the internet link that provided access to the informed consent form and study questionnaires. Professional and trade organizations were identified and contacted. Website posts, e-newsletters, and social media were used to reach members of eligible occupations, with news or social media posts either by their group administrators or by the study team. The wider social media universe was reached through posts on university channels on various platforms, university-affiliated influencer posts, and a limited paid media campaign. Social media posts and outreach to organization leaders continued periodically throughout the recruitment period.

### Informed Consent

An internet link provided access to the informed consent document that described the purpose of the study, time required to complete the questionnaire, duration of the study, and options for cross-sectional or longitudinal study participation. An affirmative consent response opened the baseline questionnaire. At the end of the initial survey, participants were asked to provide their email contact information for the longitudinal study and registry, which would be used to provide them with future questionnaires and information about studies for which they might qualify. The consent form also stated that information would be used only for medical research and shared only with other medical researchers. Participants were informed that they could end participation at any time.

### Registration

CHAMPS has been registered at ClinicalTrials.gov (NCT04370821).

### Measures

The baseline questionnaire consisted of questions related to the participants’ demographic information, occupation, and work environment, followed by validated instruments on behavioral health, described below. Instruments were selected to balance validity and brevity. An open-ended question provided an opportunity to describe personal experiences of working during the COVID-19 pandemic and participants’ interest in being interviewed. The measures and timepoints of data collection are outlined in Table 1.

**Table 1:** COVID-19 Study of Healthcare and Support Personnel (CHAMPS) measures and data collection timepoints. All data are self-reported.

Measure	Instrument	Items, n	T0 (baseline)	T1 (6 months)	T2 (Year 1)	T3 (Year 2)	T4 (Year 3)	T5 (Year 4)
Demographics	N/A^a^	N/A	✓					
Anxiety	GAD-7^b^	7	✓		✓	✓	✓	✓
Depression	PHQ-2^c^	2	✓		✓	✓	✓	✓
Social support	OSSS-3^d^	3	✓					
Sleep	ISI-7^e^	7	✓		✓	✓	✓	✓
Traumatic stress	IES-R^f^	22	✓		✓	✓	✓	✓
Stress specific to COVID-19	Based on Wu et al [4] and Chong et al [5]	10	✓					
Resilience	BRS^g^	6		✓	✓	✓	✓	✓
Proactive Coping	Proactive Coping Subscale of the PCI^h^	14		✓				
Burnout	OLBI^i^	16		✓	✓	✓	✓	✓
Eating habits	LOCES^j^	7		✓	✓	✓	✓	✓
Morphometric	Height, weight	2		✓				
Alcohol and substance abuse	N/A	2		✓				
Physical activity	N/A	2		✓				
Health history checklist	N/A	14		✓	✓	✓	✓	✓

^a^N/A: not applicable.

^b^GAD-7: Generalized Anxiety Disorder-7.

^c^PHQ-2: Patient Health Questionnare-2.

^d^OSSS-3: Oslo Social Support Scale-3.

^e^ISI-7: Insomnia Severity Index-7.

^f^IES-R: Impact of Events Scale - Revised.

^g^BRS: Brief Resilience Scale.

^h^PCI: Proactive Coping Inventory.

^i^OLBI: Oldenburg Burnout Inventory.

^j^LOCES: Loss of Control Over Eating Scale.

#### Demographic Data, General Health Status, and SARS-CoV-2 Infection

These will be ascertained by specific questions related to age, sex, occupation, self-reported general health, and infection status at the time of enrollment.

#### Anxiety and Depression

Anxiety symptoms will be assessed by the Generalized Anxiety Disorder-7 (GAD-7) [33], and depressive symptoms will be assessed by the Patient Health Questionnaire-2 (PQH-2) [19]. The GAD-7 was validated on 2740 adults, of whom 965 were interviewed by a mental health professional. The PHQ-2 includes the first 2 items of the PHQ-9, whose sensitivity and specificity are associated with the prevalence of depression [34]. These instruments were selected for their performance and brevity.

#### Social Support

The Oslo Social Support Scale-3 (OSSS-3) [35] will be used to measure social support. We sought a brief social support scale with good psychometric properties. With a Cronbach α of .640, the 3-item OSSS has good internal consistency and acceptable construct validity [35], especially for a short scale. It is scored easily, with normative values available for men and women between the ages of 14 and 91. In a cohort of adults 50 to 69 years of age who experienced adverse childhood events, moderate to strong perceived social support scores as measured by the OSSS-3 were associated with significantly lower odds of depressive symptoms [36].

#### Sleep Quality

The Insomnia Severity Index-7 (ISI-7) will be used to measure sleep quality. The ISI-7 has been validated as a useful clinical tool to quantify perceived insomnia severity for primary as well as secondary insomnia and in young and older patients [37]. It has also been validated for web-based applications, as the psychometric properties of the web-based version were found to be similar to those of the form version [38]. In a study of medical staff in China following the COVID-19 outbreak, ISI-7 scores were associated with anxiety levels and isolation and moderated by level of education [39].

#### Stress

Stress has been associated with elevated risk of cardiovascular, metabolic, and musculoskeletal disorders [18]. Essential workers may experience stress from numerous sources that are not necessarily related to the pandemic. These may interact with stressors that are related to COVID-19 work life, suggesting that both general stress and stress specific to COVID-19 should be ascertained. We will measure *traumatic stress* with the Impact of Events Scale - Revised (IES-R) [40], which assesses the symptoms of PTSD. For *stress specific to COVID-19*, we adapted scales from Wu et al [4] and Chong et al [5] to assess the work environment and concerns specifically related to working in a high-risk environment.

#### Narrative Response Question

The last question on the questionnaire provides the participants with an opportunity to describe their service experiences during the pandemic in narrative form for qualitative analysis, with no limits on the time or length of the narrative.

#### Six-Month Follow-up

The first follow-up occurred 6 months after enrollment and completion of the baseline questionnaire. Assessments included resilience, burnout, eating habits, and alcohol and substance use; physical activity; nutrition; height, weight, and weight discrimination items [41,42]; and a comprehensive health history checklist.

#### Resilience

Resilience will be measured by the Brief Resilience Scale (BRS), which is designed to assess the ability to bounce back from stress and to be brief [43]. It includes 3 positively and 3 negatively worded items, which is thought to reduce the effects of social desirability and positive response bias. This scale is unique because it assesses resilience rather than the attributes that contribute to resilience. Whether any aspects of resilience in middle life predict long-term health outcomes after major life events such as the COVID-19 pandemic remains to be determined.

#### Proactive Coping

Proactive coping is defined as a forward-looking strategy to address anticipated stressors or hardships, and it will be measured by the Proactive Coping Subscale of the Proactive Coping Inventory (PCI). We hypothesized that proactive coping would serve as a moderator of the consequences of stress. The Proactive Coping Subscale of the PCI consists of 14 items that combine autonomous goal setting with self-regulatory goal attainment cognitions and behavior. The scale showed high internal consistency, as seen in reliability measures of .85 and .80 in two samples during its development [44,45].

#### Burnout

Burnout will be assessed by the Oldenburg Burnout Inventory (OLBI). Burnout is a psychological condition that follows highly demanding or stressful physical and mental work conditions that may be accompanied by inadequate resources [15,46]. Historically, the most commonly used instrument is the Maslach Burnout Inventory [47], which has been criticized on psychometric grounds because all items in each subscale are unidirectional [15]. We chose the OLBI because it avoids this problem by including positively and negatively framed items in the assessment of exhaustion and disengagement, which are two core features of burnout [15]. Moreover, the OLBI includes a stronger assessment of the cognitive aspect of work, which plays an important role in numerous health care functions. Data from the OLDI are also available from previous studies of health care workers [15].

#### Eating Habits

Increased caloric intake is a common response to mental stress [48]. We chose the brief, 7-item Loss of Control Over Eating Scale (LOCES) [49] to evaluate eating habits, as it maintains a good fit to the data analyzed by the original, 24-item LOCES [50]. The quality of the diet is as important as the caloric intake. We used a 2-question assessment of fruit and vegetable intake that has high specificity, based on correlations with biomarkers in the test population (plasma ascorbic acid, beta carotene and alpha tocopherol 24-hour urinary potassium excretion) [51].

#### Morphometrics

Self-reported height and weight were obtained. Weight discrimination history, namely whether the participant experienced any bias based on body weight, was assessed by a single question [41,42].

#### Alcohol and Substance Use

We hypothesized that alcohol and substance use may increase in response to the stress of workplace demands, but we thought that a thorough screening would not be in the best interest of the overall study. We decided to limit our data to answers to two questions, namely, whether the participants’ alcohol and substance use increased, was unchanged, or decreased since the start of the pandemic.

#### Physical Activity

We chose to assess physical activity with two brief questions: one on the amount of time the participants spent in moderate-to-vigorous physical activity, and one on whether their amount of physical activity had changed since the pandemic.

#### Health History Checklist

The relationship among stress, major life events, and other biopsychosocial factors has a long history of study in epidemiology and public health, with a sound mechanistic basis [52,53,54]. We hypothesized that the effects of working in the challenging circumstances of the COVID-19 pandemic would have effects on health over time. We obtained a comprehensive history of the participants’ mental and physical health during the pandemic to enable prospective evaluation of incident conditions.

### Exposure Assessment

We will define and quantify exposure to COVID-19 service burden by summing participants’ scores on the Perception of Stress at Work scale developed by Wu and colleagues [4] in 2009 during the SARS pandemic, and we will add a point to that score for each of several additional stressors: (1) working more hours since the pandemic; (2) working in a different setting; (3) caring for patients with COVID-19; (4) changing one’s living arrangement; (5) receiving a COVID-19 diagnosis; and (5) having a close contact (friend, family member, colleague, or any loved one) with a COVID-19 diagnosis (Table 2).

For this purpose, perceived stress was deemed to be an outcome of exposure, but it can also be included as an exposure variable in models that ascertain incidence of health conditions over time.

**Table 2:** Components of the algorithm-based score for exposure to COVID-19 service burden.

Component	Scoring
Perception of Stress at Work scale	Score of 9-item scale
Working more hours	Yes=1 additional point
Working in a different setting	Yes=1 additional point
Caring for patients with COVID-19	Yes=1 additional point
Changing one’s living arrangement	Yes=1 additional point
Receiving a COVID-19 diagnosis	Yes=1 additional point
Having close contact (friend/family member/colleague/any loved one) with a COVID-19 diagnosis	Yes=1 additional point

### Annual Follow-up Questionnaires

Annual follow-up questionnaires will include incidence of conditions and diseases, as well as selected instruments from the baseline and 6-month follow-up questionnaires. Additional items may be added in response to the evolving research literature on COVID-19 and that in other fields. Table 1 provides the anticipated data collection schedule.

### Registry and Ancillary Studies

During enrollment participants were given the option of providing contact information for follow-up questionnaires, which also enrolls them in the CHAMPS Registry. The CHAMPS Registry enables recruitment of eligible individuals for clinical trials of interventions relevant to COVID-19 or other disorders or conditions, as well as for ancillary studies of basic behavioral research or observational studies that require new data collection. Anticipated ancillary studies include a detailed assessment of lifestyle factors, interactions with family life, and wellness interventions. Ancillary study proposals will be reviewed by the CHAMPS Steering Committee for relevance, scientific interest, and participant burden.

### Analysis

#### Data Management and Quality

Data are collected through the web-based Qualtrics system. SPSS (IBM Corporation) and SAS (SAS Institute, Inc) will be used for data analysis. Initially, we will perform an exploratory analysis to establish patterns of missingness and distributions of the variables being measured. The registry data will be characterized with descriptive analyses, including frequency distributions, prevalence rates of COVID-19 testing and SARS-CoV-2 infection, and means and standard deviations of scores on the scales measuring work environment, general health status, anxiety, depressive symptoms, social support, traumatic stress, social support, sleep quality, and stress. Multiple logistic and linear regression methods will be used to assess baseline associations of health and well-being with estimates of exposure to COVID-19 infection and COVID-19–related stress at work. Models will be adjusted for sex, age, race/ethnicity, and job classification. Other preliminary explorations of associations between measured variables will be performed to aid the development of substudies from the registry. Follow-up health status questionnaires will enable analysis of associations between incident health events and baseline variables.

Narrative responses to the final question on the questionnaire will be analyzed using a deductive approach and thematic content analysis techniques. Multiple investigators will read the narratives provided by participants to perform coding of the significant statements made by participants. After multiple readings, investigators will generate data categories and clusters of statements and will develop themes.

#### Aim 1

We will determine whether essential workers with higher COVID-19 service exposure burden will experience higher rates of stress, depression, anxiety, and substance use than the general population at the time they enroll in CHAMPS, and higher than in similar populations before the COVID-19 pandemic. We will compare exposure by type of service, occupation, and place of service (eg, clinical setting or community), controlling for age, self-reported sex, and race. We will conduct analyses to identify mediators, such as racial status, and moderators, such as proactive coping and resilience.

#### Aim 2

We will determine whether the severity of mental health symptoms will be associated with changes in the prevalence of SARS-CoV-2 infections by geographic region.

#### Aim 3

We will ascertain whether exposure to COVID-19 service burden will affect physical and mental health over time and how these are influenced by psychosocial variables (eg, stress, depression, anxiety, burnout). We will analyze whether longitudinal health outcomes are associated with type of service; severity and duration of mental health symptoms; and age, sex, race, and other variables. We anticipate that some analyses will depend on the adequacy of the sample sizes in various demographic subgroups.

#### Aim 4

We will obtain longitudinal data to determine whether mental health symptoms will persist longer among participants with more severe symptoms and whether these symptoms will be associated with higher incidence of physical health conditions.

#### Aim 5

We will ascertain the extent to which the thematic contents of the narrative responses to the final question of the questionnaires are correlated with measures of distress (stress, anxiety, depression, burnout) and moderated by measures of resilience. We will ascertain whether thematic content is a moderator or mediator of health outcomes and is associated with retention of participants over time.

## Results

A review of the published literature on relationships between stressful life events and health led to the hypothesis that stress experienced by essential workers during the COVID-19 pandemic would affect their health status over time. In response, CHAMPS was designed to ascertain work-life experience during the first year of the pandemic, exposure to stress, and self-reported mental and physical health, as well as to determine effects of service during the pandemic on health over the subsequent 5 years. The CHAMPS longitudinal cohort serves as a registry for recruiting participants in future ancillary studies. After IRB approval, 2762 participants were enrolled between May 2020 and June 2021, with 1534 providing contact information for future studies and follow-up questionnaires.

## Discussion

### Principal Considerations

The longitudinal CHAMPS Study and Registry will provide important prospective data about relationships between extended health care–related service during a global pandemic and the future health of the health care–related workforce. Of special interest are first responders and service workers, who are less often included in such studies, as well as a broad ethnic diversity of participants. The lengthy enrollment period as the pandemic progresses should result in a sample with a wide range of exposures to various working conditions, thereby providing insights into the potential attributes that contribute to changes in health over time. Assessments of an extensive range of psychosocial factors, such as lifestyle habits, resilience, and pre-existing conditions, will enable assessment of their influence on mental and physical health.

The CHAMPS Registry is designed to provide opportunities for investigating additional questions and potential extension of data collection for specific hypotheses. The CHAMPS Registry anticipates ancillary studies of behavioral and biological mechanisms of action, studies of lifestyle changes and health, the effects of the pandemic on extended families, and potential randomized trials of interventions.

Altogether, CHAMPS has the potential to provide advances in knowledge about the role of chronic stress in health. The value of the study for some questions, such as identification of associations with various demographic variables, depends on the availability of a sufficient sample size of participants. We anticipate that over time, the study findings will inform public health policy relevant to national health emergencies.

### Limitations

This is a convenience sample, which limits the generalizability of the results to the entire population of essential workers. Some occupation categories will have small sample sizes; thus, we may not have sufficient representation for individual analyses. Self-selection bias may also be a factor if some individuals decline to participate due to reluctance to be perceived as not supportive of their institutions. The measurement of COVID-19 service exposure relies on an algorithm that should be evaluated against an independent data set. Finally, the planned longitudinal analyses will depend on retaining a sufficient proportion of participants over time.

## Abbreviations

BRS: Brief Resilience Scale
CHAMPS: COVID-19 Study of Healthcare and Support Personnel
GAD-7: Generalized Anxiety Disorder-7
IES-R: Impact of Events Scale - Revised
IRB: Institutional Review Board
ISI-7: Insomnia Severity Index-7
LOCES: Loss of Control Over Eating Scale
OLBI: Oldenburg Burnout Inventory
OSSS-3: Oslo Social Support Scale-3
PCI: Proactive Coping Inventory
PHQ-2: Patient Health Questionnaire-2
PTSD: posttraumatic stress disorder
SARS: severe acute respiratory syndrome
